# Multiarticular Deforming and Erosive Tophaceous Gout With Severe Comorbidities

**DOI:** 10.1097/RHU.0000000000001121

**Published:** 2019-06-13

**Authors:** Aurelian Anghelescu

**Affiliations:** From the Neurorehabilitation Clinic, Teaching Emergency Hospital “Bagdasar-Arseni,” Bucharest, Romania.

Gout is an increasingly common chronic metabolic disorder, resulting from the inflammatory responses to monosodium urate crystals deposited in tissues.

Tophaceous gout can occur years after recurrent attacks of acute inflammatory arthropathies. Microscopically, the tophus (the cardinal feature of advanced gout) is a foreign body, granuloma-like structure that contains lumps of monosodium urate crystals and is surrounded by inflammatory cells and connective tissue.^1^

Multiarticular chronic tophaceous gout causes progressive joint damage and the development of multiple, severe destructive ulcerations and reduces the quality of life.

Although a few cases of erosive polyarticular tophaceous gout with severe osteolysis that require digital amputation have been identified in the literature, none of these have demonstrated extensive bone destruction.

A 57-year-old white man with chronic tophaceous multiarticular gout was referred to our neurorehabilitation clinic.

He presented with tophi on the right pinna and left knee, “white bumps” on the left elbow, multiple ulcerated tophi on his feet and hands, and necrotic lesions (distal right thumb and the distal phalanx of the little finger of the right hand). He was recently treated with vancomycin for a methicillin-resistant *Staphylococcus aureus* infection.

He had a medical history of heavy smoking, longstanding and suboptimally treated gout (since 2004), previous partial amputation of the fourth right finger (2015), arterial hypertension (since 2012), renal lithiasis (since 2014), moderate kidney disease, myocardial infarction and coronary stent (2012), left sylvian ischemic stroke, and right hemiplegia and aphasia (in December 2018).

**FIGURE 1:**
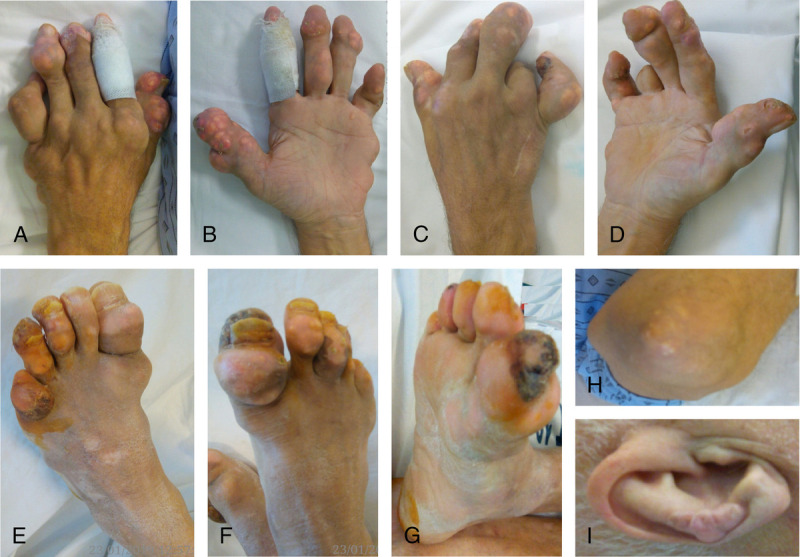
A and B, Left hand. C and D, Right hand. E, Left foot. F and G, Right foot. H, Left elbow. I, Right pinna.

**FIGURE 2:**
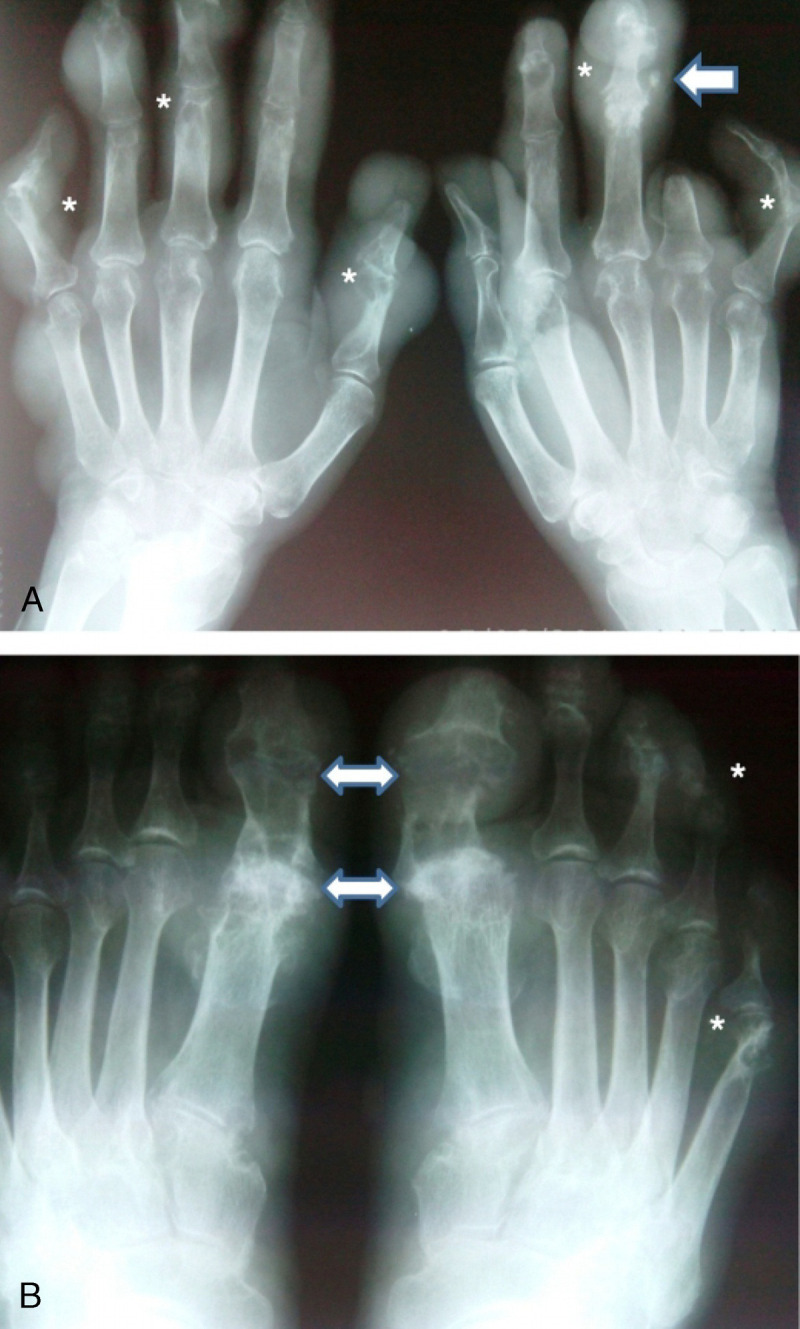
A, Anteroposterior radiograph of the hands: calcified tophus (arrow), suggesting renal impairment. Multiple bilateral interphalangeal articular erosions, adjacent to tophus (asterisk). B, Anteroposterior radiograph of the feet: multiple bilateral interphalangeal (asterisk) and metatarsophalangeal joints erosions (left-right arrow).

Local physical and radiologic examination shows multiarticular deformities and deviations of the fingers and toes, soft-tissue involvement (multiple tophi, erosive and necrotic lesions; Fig. 1, Fig. 2).

Laboratory testing revealed uric acid (UA) 7.5 mg/dL (446 μmol/L), erythrocyte sedimentation rate 130 mm/h, white blood cells 6300/μL, 65.6% neutrophils, plasma fibrinogen 854 mg/dL, C-reactive protein 20.2 mg/L, and serum creatinine 1.70 mg/dL (glomerular filtration rate 44 mL/min per 1.73m^2^).

Patient's participation in the rehabilitation program was limited by his dysfunctional medical comorbidities and neurologic status.

Lifetime diet, long-term colchicine 0.5 mg twice daily and allopurinol 300 mg twice daily, neurotrophic factors, antihypertensive, antiplatelet therapy, and follow-up were indicated at discharge.

Gout represents a risk factor for multiple complications, including chronic kidney disease, diabetes mellitus, hypertension, dyslipidemia, and increased mortality.^1^

Gout is a risk factor for cardiocerebrovascular disease, compared with diabetes for incident ischemic stroke.^2^

Although UA is the primary endogenous antioxidant and free radical scavenger in the blood and is potentially neuroprotective during acute ischemic stroke, patients with cerebral ischemia and high UA levels (>380 μmol/L) or low UA levels (≤250 μmol/L) are 2 to 3 times more likely to have a poor outcomes than those with normal UA levels (250–380 μmol/L).^3^

The current standard of care for gout patients with accumulated comorbidities and chronic health problems requires a multidisciplinary/interdisciplinary team management approach.

Due to the favorable pharmaceutical control of hyperuricemia, the surgical treatment of tophaceous ulcerated gout is seldom necessary and is indicated only in severe cases that present with joint destruction, deformities, and infection.^4^

Gout management requires long-term, urate-lowering therapy to achieve a serum urate concentration of less than 5 mg/dL (300 μmol/L)^1^

Allopurinol, in combination with aspirin and lipid-lowering therapy, may improve endothelial function and inflammation and may reduce the occurrence of disabilities in patients with stroke.^5^
